# Corrosion inhibition mechanisms of metal-organic frameworks in ammonia-rich environments

**DOI:** 10.3389/fchem.2025.1644300

**Published:** 2025-09-22

**Authors:** Jiao-Jiao Cao, Yu-Meng Wu, Jin-Long Ge, Qing-Min Yang, Zhen-Yu Chen

**Affiliations:** 1 Engineering Technology Research Center of Silicon-based Materials, School of Material and Chemical Engineering, Bengbu University, Bengbu, China; 2 Key Laboratory for Material Chemistry of Energy Conversion and Storage, Ministry of Education, School of Chemistry and Chemical Engineering, Huazhong University of Science and Technology, Wuhan, China

**Keywords:** MOFs, corrosion inhibition, ammonia-containing aqueous environments, copper, ZIFs

## Abstract

A range of metal-organic framework (MOF)-based composite materials were synthesized and assessed for their corrosion inhibition properties in ammonia-containing aqueous environments. The interaction mechanisms of these materials with copper surfaces were systematically investigated using electrochemical techniques and surface characterization methods. Based on these analyses, a comprehensive mechanistic model was developed to explain the interplay of the observed factors. The results demonstrated that the corrosion inhibition performance of zeolitic imidazolate frameworks (ZIFs), a representative class of MOFs, is significantly influenced by the surrounding environment. Specifically, experimental analysis revealed a competitive interaction between NH_3_ and the ZIF ligand in complex reactions, leading to structural instability of the ZIFs. This instability compromises the protective layer formed on the copper surface, resulting in a reduction of up to 60% in corrosion inhibition efficiency and, consequently, insufficient long-term durability.

## Introduction

During prolonged use, metal materials are often subject to inevitable corrosion, which significantly reduces their service life and hinders their performance in demanding industrial applications. The fundamental process of metal corrosion involves the oxidation of metal atoms, which lose electrons to form positively charged metal ions (Ali et al., 2024; Mussttaf et al., 2024; Privitera et al., 2024). The prevalence of corrosion problems can lead to severe environmental pollution and may even trigger catastrophic events such as oil and gas leaks, bridge collapses, civil aviation disasters, and the loss of cultural heritage. Literature estimates the annual economic loss due to corrosion to be approximately 3.4% of the global GDP. Statistics indicate that the implementation of effective corrosion protection strategies could save between 15% and 35% of global corrosion costs (González-Parra and Di Turo, 2024; Wang et al., 2024; Yousif et al., 2024). Currently, common methods of corrosion protection include surface treatments, structural modifications, electrochemical protection, and the addition of corrosion inhibitors. Among these, the addition of corrosion inhibitors is a widely adopted method for mitigating metal corrosion due to its cost-effectiveness and ease of implementation (Cunha et al., 2024; Moumouche et al., 2024; Zhuang et al., 2025).

Corrosion inhibitors are substances that, when added in small amounts to a corrosive medium, provide protection against metal corrosion (Fattah-alhossein et al., 2024; Yang S. et al., 2024; Zhang et al., 2024). Extensive research has investigated the effects of various compounds on metal corrosion in different corrosive environments. Organic compounds containing heteroatoms such as nitrogen, oxygen, sulfur, or phosphorus, or possessing unsaturated bonds, have demonstrated remarkable corrosion inhibition properties (Abtan et al., 2024; Milkov et al., 2024; Sherif and Park, 2006). Their mechanism of action involves strong interactions between the organic compounds and the metal substrate, thereby protecting against metal corrosion. Traditional corrosion inhibitors are widely used and supported by an evolving theoretical framework. However, these traditional inhibitors often possess biological and environmental toxicity, making the design and development of biocompatible and environmentally friendly corrosion inhibitors a significant research focus. To address these limitations, emerging materials like metal-organic frameworks (MOFs) have gained attention. MOFs are a class of organic-inorganic hybrid compounds with tunable structures formed through the coordination between metal clusters and organic ligands. They are characterized by their large specific surface area, chemical stability, and adjustable framework structures, which have led to their widespread application in catalysis, drug delivery, gas adsorption, and metal corrosion prevention, among other fields (Shin et al., 2024; Xiong et al., 2024; Yang C. et al., 2024). Zeolitic imidazolate frameworks (ZIFs) represent a prominent subclass within the broader family of metal-organic frameworks (MOFs). The effectiveness of corrosion inhibitors hinges significantly on their inherent molecular characteristics, which, to a large extent, govern the stability of inhibitor molecule adsorption on the metal surface (Yang et al., 2025; Zhang D. et al., 2023; Zhang et al., 2025). Prior research indicates that organic molecules used as corrosion inhibitors are heterocyclic compounds or contain polar functional groups (Pham et al., 2024; Shin et al., 2024; Singh, 2024; Subramaniyan et al., 2024). Environmental factors such as temperature, pH, and the presence of complex ions can also impact corrosion inhibition performance. Ions with strong complexing abilities, such as NH^4+^ and NH_3_ (Cai et al., 2021; Ruhl and Jekel, 2013; Tasic et al., 2016), present in the corrosive medium, are significant factors affecting the corrosion inhibition performance of inhibitors, as they can react with the corrosion inhibitor, reducing its effective adsorption on the metal surface and leading to a decline in corrosion inhibition efficiency.

Particularly in copper-based systems, long-term exposure to ammonia-containing solutions can result in heightened stress corrosion sensitivity, with the potential for localized chemical reactions such as galvanic corrosion, pitting corrosion, and erosion-corrosion (Narváez-Romo et al., 2017; Wirtz et al., 2022). Furthermore, ammonia can act as a complexing agent, forming complexes with elements like Ag^+^, Co^2+^, Ni^2+^, and Zn^2+^ (Helios et al., 2010; Liang et al., 2007; Potemkin et al., 2019). Given that MOFs contain a multitude of heterocyclic organic groups including nitrogen, oxygen, and sulfur, many researchers are dedicated to developing MOFs with superior corrosion inhibition performance. Additionally, due to MOFs’ characteristics of multiple pores, large specific surface area, and numerous adsorption sites, some studies have focused on utilizing MOFs as carriers for corrosion inhibitors to achieve intelligent, controlled release of corrosion inhibitor molecules. However, when copper metal is used in other corrosive environments, such as acidic or basic conditions, it is essential to understand how MOFs behave in solution, how their structures change under these conditions, and how their corrosion inhibition properties are affected. These critical knowledge gaps motivate systematic investigation of the behavior of MOFs in corrosive environments.

In this study, the impact of ammonia on the corrosion inhibition properties of Zn-ZIF, Co-ZIF, and Ni-ZIF, which contain different metal center ions, was investigated. The mechanisms underlying the influence of ammonia on the corrosion inhibition properties of these ZIFs were elucidated using electrochemical testing, SEM, TEM, FTIR, and XRD analyses.

## Experimental materials and characterization

### Experimental materials

The metallic material employed in the experiments was T2 copper. The chemicals utilized included zinc nitrate hexahydrate, cobalt nitrate hexahydrate, nickel nitrate hexahydrate, and 2-methylimidazole. The solvents used were methanol and ammonia, both of which were of analytical grade and did not require further purification.

The ZIFs were synthesized from The Zn-MOFs were synthesized from zinc nitrate hexahydrate, cobalt nitrate hexahydrate, nickel nitrate hexahydrate and 2-amino-benzimidazole under solvothermal conditions.

### Test characterization and methods

X-ray diffraction (XRD) is an analytical technique employed for investigating crystal structures. By measuring characteristic diffraction peaks of crystals, XRD enables qualitative phase analysis of materials. In this study, XRD was primarily utilized to analyze the structural characteristics of synthesized ZIFs powders with different metal center ions, thereby determining their crystalline phase composition and structural configuration. The synthesized materials were characterized using a suite of analytical techniques, including Scanning Electron Microscopy (SEM), Transmission Electron Microscopy (TEM), and Infrared Spectroscopy. SEM was employed to obtain detailed surface morphology and size distribution of the samples, providing valuable insights into the material’s microstructure. TEM offered high-resolution imaging, revealing the intricate structural details of the samples. Infrared spectroscopy was utilized to identify the molecular structure and chemical bonding within the samples by analyzing the characteristic wavelengths associated with specific chemical bonds, serving as a powerful tool for substance characterization and identification.

Electrochemical tests were conducted on the metal surfaces post-treatment with the synthesized materials using an electrochemical workstation. These tests included open-circuit potential measurements, electrochemical impedance spectroscopy, and potentiostatic polarization. Open-circuit potential testing facilitated the assessment of the material’s potential state in the absence of current flow, offering insights into its equilibrium electrochemical state. Electrochemical impedance spectroscopy allowed for the analysis of the charge transfer processes and mass transfer limitations at the electrode interface, providing a comprehensive understanding of the electrochemical behavior. Potentiostatic polarization testing was employed to investigate the current-time response of the materials at a fixed potential, which is essential for elucidating the electrochemical stability and corrosion inhibition characteristics of the materials. Through these electrochemical analyses, the influence of the synthesized materials on the electrochemical properties of the metal surfaces was thoroughly investigated.

## Results

**FIGURE 1:**
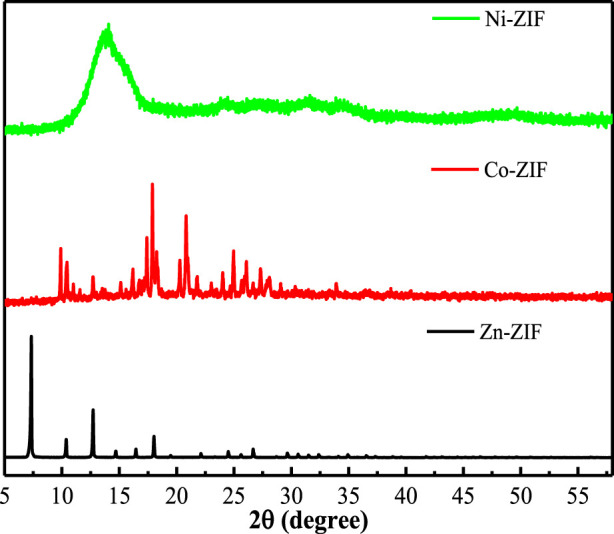
XRD patterns of ZIFs with metal center ions.

ZIFs characterization and results. XRD characterization was performed to analyze the crystallinity of ZIFs with different metal centers (Figure 1). The diffraction patterns revealed distinct crystalline structures for each variant. Zn-ZIF displayed characteristic Bragg reflections at 2θ = 5.16°, 10.52°, 12.78°, 14.92°, 16.64°, 18.24°, 24.66°, 26.90°, 28.22°, 31.37°, and 33.33° (Aboraia et al., 2020; Liu et al., 2015), consistent with its reported crystalline structure. Co-ZIF exhibited diffraction peaks at 2θ = 7.4°, 10.4°, 12.7°, 14.8°, 16.5°, 18.0°, 22.1°, 24.5°, 25.5°, 26.7°, 29.5°, 30.6°, and 32.5° (Guo et al., 2016; Liu et al., 2021), confirming successful formation of the Co-based framework. Ni-ZIF demonstrated three prominent peaks at 2θ = 9.0°, 15.7°, and 16.8° (Yang and Bai, 2019), indicating its distinct crystalline arrangement compared to Zn and Co analogs.

**FIGURE 2:**
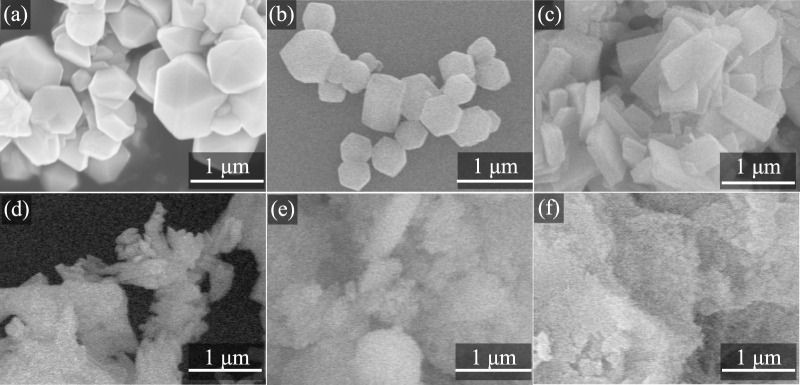
SEM images of ZIFs treated with NH_3_·H_2_O at 25 °C: before **(a)** Zn-ZIF **(b)** Co-ZIF **(c)** Ni-ZIF and after **(d)** Zn-ZIF (NH_3_·H_2_O) **(e)** Co-ZIF (NH_3_·H_2_O) **(f)** Ni-ZIF (NH_3_·H_2_O).

The pristine Zn-ZIF, Co-ZIF, and Ni-ZIF samples, characterized via scanning electron microscopy (SEM) and transmission electron microscopy (TEM), were immersed in a 20 mg/L ammonia solution to assess their structural stability. Prior to ammonia exposure, SEM images (Figures 2a–c, Scale bar: 1 μm) revealed that all three ZIFs exhibited a uniform rhombic dodecahedral morphology, demonstrating monodispersity and well-defined crystallinity. However, following a 24-h immersion period in the ammonia solution (Figures 2d–f, Scale bar: 1 μm), SEM analysis indicated a significant structural disintegration, with the ZIFs transforming into irregular fragments, suggesting a collapse of the original framework. This structural degradation is likely attributable to the coordination of ammonia molecules with the metal centers within the ZIFs, leading to disruption of the inherent framework topology.

**FIGURE 3:**
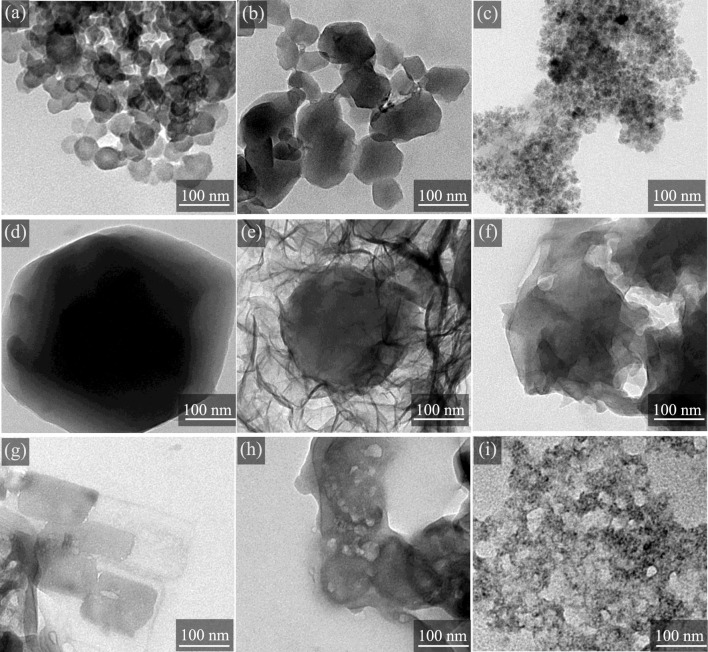
TEM images of different metal center ions-ZIFs treated with 20 mg/L NH_3_·H_2_O at 25 °C: before **(a)** Zn-ZIF **(d)** Co-ZIF **(g)** Ni-ZIF and after **(b,c)** Zn-ZIF (NH_3_·H_2_O) **(e,f)** Co-ZIF (NH_3_·H_2_O) **(h,i)** Ni-ZIF (NH_3_·H_2_O).

Transmission Electron Microscopy (TEM) was used to examine the micromorphological changes in Zn-ZIF, Co-ZIF, and Ni-ZIF after exposure to ammonia. TEM images (Figure 3a for Zn-ZIF, Figure 3d for Co-ZIF, and Figure 3g for Ni-ZIF) show that the ZIFs, distinguished by their metal center ions, exhibit angular, regular shapes and are uniformly dispersed. However, within the ammonia environment (Figures 3b,c,e,f,h,i), the ZIFs initially show signs of damage, with blurred boundaries, eventually disintegrating into fragments. This observation suggests that ammonia exposure induces framework collapse through ligand displacement, as evidenced by particle fragmentation in TEM.

**FIGURE 4:**
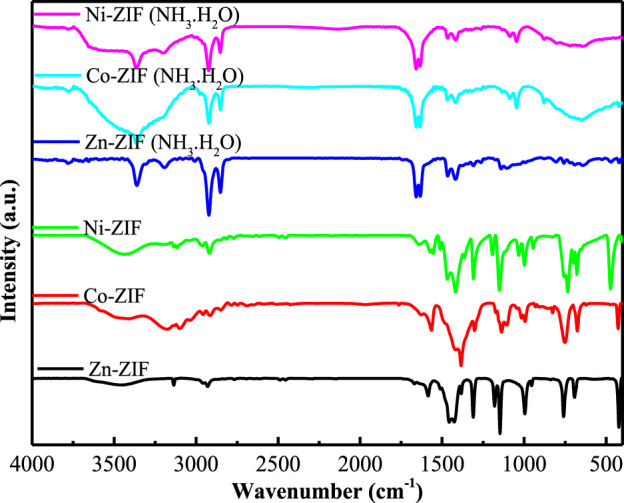
FTIR of different metal center ions-ZIFs treated with ammonia at 25 °C.

The potential degradation of the microscopic morphology of Zn-ZIF, Co-ZIF, and Ni-ZIF in an ammonia environment may stem from the formation of coordination complexes between the metal ions within the ZIFs’ structure and ammonia molecules. To verify this hypothesis regarding coordination complex formation, FTIR analysis was subsequently performed to track the chemical bonding changes. We conducted FTIR analysis of Zn-ZIF, Co-ZIF, and Ni-ZIF to gain a deeper understanding of their structural transformations. As illustrated in Figure 4, the intense band at 422 cm^-1^ in the Zn-ZIF absorption spectrum indicates the Zn-N stretching mode. Similarly, the strong band at 426 cm^-1^ in the Co-ZIF spectrum corresponds to the Co-N stretching mode, while the absorption peak at 472 cm^-1^ in the Ni-ZIF spectrum is characteristic of Ni-N stretching.

After ammonia treatment, the characteristic peaks associated with nitrogen and metal ions vanish from the ZIFs’ spectra, and new peaks emerge at 3,367 cm^-1^ (Zn-ZIF), 3,352 cm^-1^ (Co-ZIF), and 3,366 cm^-1^ (Ni-ZIF), respectively. The disappearance of metal-nitrogen bonds combined with the emergence of NH_3_ vibrations strongly indicates ammonia molecules are displacing the original ligands, which necessitates re-examination of the metal coordination geometry. These new peaks are attributed to the stretching vibration of the intrinsic complex NH_3_ (Breternitz et al., 2018; Wang et al., 2020). This observation aligns with the correlation between the valence orbital hybridization mode of metal ions and infrared wavenumbers. Zn^2+^, Co^2+^, and Ni^2+^ are identified as six-coordinate metal ions, contrasting with their original four-coordinate geometry in ZIF frameworks, typically adopting an sp^3^d^2^ hybridization configuration.

### Assessment of the impact of NH_3_·H_2_O on the corrosion inhibition efficacy of ZIFs

**FIGURE 5:**
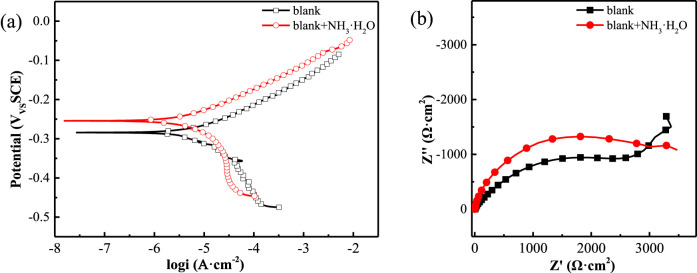
Electronichemical measurements of copper in 0.5 mol/L NaCl solution with NH_3_·H_2_O at 25 °C **(a)** polarization curves **(b)** Nyquist plots.

**FIGURE 6:**
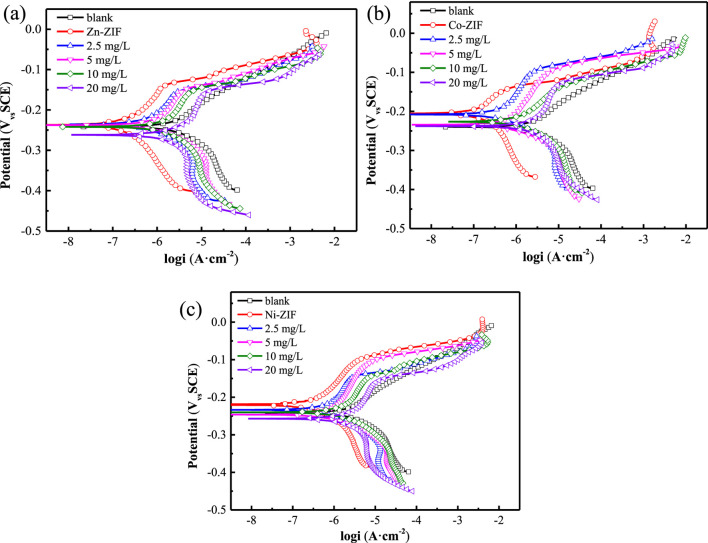
Polarization curves of copper in 0.5 mol/L NaCl solution with ZIFs and NH_3_·H_2_O at 25 °C **(a)** Zn-ZIF **(b)** Co-ZIF **(c)** Ni-ZIF.

To establish a comparative baseline, we first conducted electrochemical tests to assess the corrosion behavior of copper in a 0.5 M NaCl solution without any additives (Figure 4). In the polarization curve (Figure 5a), the addition of 8.2 mM ammonia resulted in a minor positive shift in the corrosion potential, with the corrosion current density remaining virtually unchanged. This suggests that the inhibitory effect on the metal surface corrosion was not pronounced. Examination of the Nyquist plot (Figure 5b) revealed a slight expansion in the impedance arc diameter when ammonia was present, suggesting negligible influence of aqueous ammonia on the corrosion behavior of copper under these conditions. After establishing the baseline corrosion behavior in ammonia-containing solution (Figure 4), we subsequently evaluated the polarization curves for a copper electrode immersed in a 0.5 M NaCl solution containing ZIFs with varying metal center ions (Figure 6). Relative to the blank condition (0.5 M NaCl without any additives), the addition of ZIFs led to an increase in the corrosion potential (*E*_*corr*_) and a decrease in the corrosion current density (*i*_*corr*_) for copper. However, with increasing ammonia concentration, *E*_*corr*_ shifted negatively and *i*_*corr*_ gradually increased, indicating that ammonia mitigates the corrosion inhibition effect of ZIFs. As detailed in Table 1, the icorr increases and the corrosion inhibition efficiency declines with rising ammonia concentration. At concentrations reaching 8.2 mM, *i*_*corr*_ values approach the blank condition levels, signifying a near-total loss of efficacy for ZIFs with different metal center ions (Aliyari et al., 2023; Zhang D. et al., 2023). This is attributed to the fact that, as ammonia concentration increases, ammonia preferentially coordinates with transition metal ions (e.g., Zn^2+^ or Co^2+^) through ligand substitution, leading to complexation reactions that disrupt the ZIFs’ structure and consequently degrade their corrosion inhibition performance.

**TABLE 1:** Polarization parameters of copper in 0.5 mol/L NaCl solution with different metal center ions-ZIFs and NH_3_·H_2_O at 25 °C.

Samples	$C_{{\text{NH}}_{3}{\cdot H}_{2}O\;}$ (mg/L)	E_corr_ (mV)	b_a_ (mV/dec)	b_c_ (mV/dec)	i_corr_ (A·cm^-2^)	IE (%)
	blank	−245	93	−129	4.56 × 10^−6^	-
	0	−234	56	−101	6.14 × 10^−7^	86.53
Zn-ZIF	2.5	−235	99	−57	1.25 × 10^−6^	72.59
	5	−237	115	−53	1.41 × 10^−6^	69.08
	10	−240	88	−61	2.38 × 10^−6^	47.81
	20	−252	64	−83	3.89 × 10^−6^	14.69
	0	−203	110	−54	4.72 × 10^−7^	89.65
Co-ZIF	2.5	−208	111	−53	8.65 × 10^−7^	81.03
	5	−216	139	−49	1.08 × 10^−7^	76.32
	10	−227	110	−54	2.45 × 10^−6^	46.27
	20	−236	83	−64	3.77 × 10^−6^	17.32
	0	−215	124	−51	9.08 × 10^−7^	80.09
Ni-ZIF	2.5	−223	111	−54	1.09 × 10^−7^	76.10
	5	−240	126	−51	1.75 × 10^−7^	61.62
	10	−243	99	−57	2.66 × 10^−6^	41.67
	20	−255	74	−70	4.09 × 10^−6^	10.31

**FIGURE 7:**
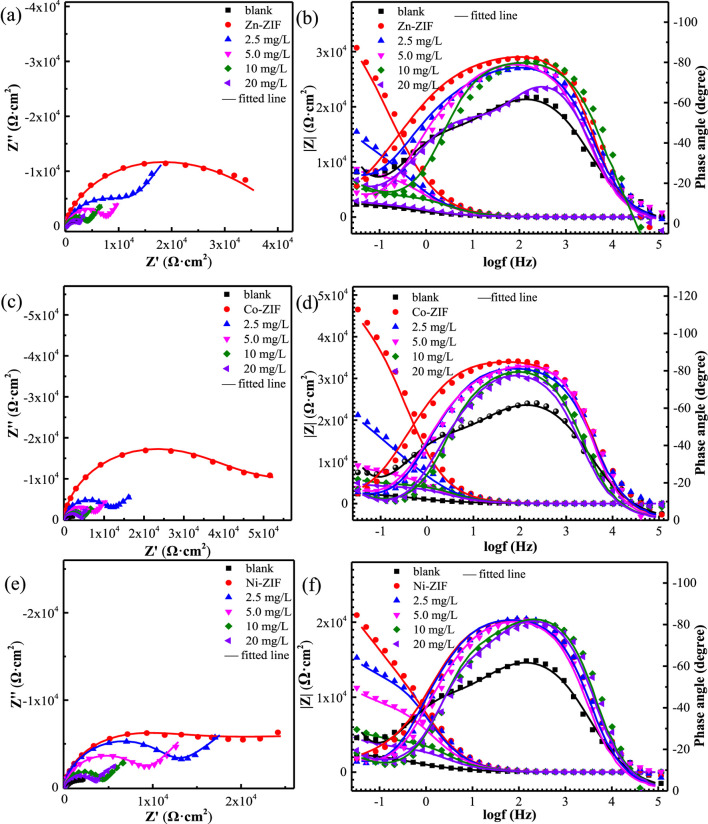
Nyquist **(a,c,e)** and Bode plots **(b,d,f)** of copper in 0.5 mol/L NaCl solution with different metal center ions-ZIFs and NH_3_·H_2_O at 25 °C: **(a,b)** Zn-ZIF **(c,d)** Co-ZIF **(e,f)** Ni-ZIF.

**FIGURE 8:**
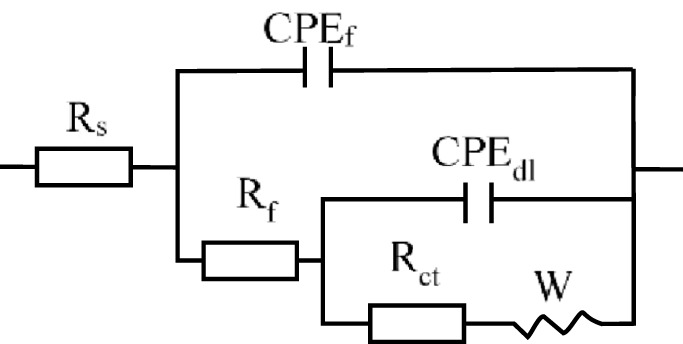
Electrochemical equivalent circuits for fitting the impedance spectra.

This study investigated the influence of ammonia on the corrosion inhibition performance of metal-core ZIFs in an ammonia-containing aqueous environment. Figure 7 showed the impedance spectra of the copper electrode. The incorporation of the ZIF variants resulted in a notable increase in the capacitive reactance, attributed to the adsorption of these ZIFs onto the copper surface. With increasing ammonia concentration from 2.5 mg/L to 20 mg/L, the capacitive reactance gradually decreased, as shown in Figures 7a,c,e. The increase in the maximum phase angle indicates the formation of a compact protective film by the metal-core ZIFs on the metal surface, effectively preventing the penetration of corrosive species, as illustrated in Figures 7b,d,f (Liu et al., 2024; Yang S. et al., 2024). However, the maximum phase angle progressively decreased with increasing ammonia concentration. The electrochemical behavior was further quantified through equivalent circuit modeling. The equivalent circuit model in Figure 8 was employed to fit the impedance data, and the corresponding parameters are detailed in Table 2. The data reveal a significant increase in both *R*_*f*_ (film resistance) and *R*_*ct*_ (charge transfer resistance) upon the addition of the ZIF variants compared to the blank conditions, indicating the formation of a dense protective film covering the electrode surface. Nevertheless, *R*_*f*_ and *R*_*ct*_ exhibited a continued decrease with increasing ammonia concentration (Bai et al., 2023; Zhang X. et al., 2023; Zhang et al., 2024).

**TABLE 2:** Fitted electrochemical parameters of the EIS for copper in 0.5 mol/L NaCl solution containing different metal center ions-ZIFs and NH_3_·H_2_O at 25 °C.

Samples	$C_{{\text{NH}}_{3}{\cdot H}_{2}O\;}$ (mg/L)	R_s_ (Ω·cm^2^)	CPE_f_-T (S^n1^·Ω^-1^·cm^-2^)	n1	R_f_ (Ω·cm^2^)	CPE_dl_-T (S^n2^·Ω^-1^·cm^-2^)	n2	R_ct_ (Ω·cm^2^)
	blank	5	8.96 × 10^−5^	0.79	526	2.01 × 10^−4^	0.67	3,025
	0	4	1.18 × 10^−5^	0.97	277	2.37 × 10^−5^	0.48	43,522
Zn-ZIF	2.5	4	1.93 × 10^−5^	0.91	2,732	3.51 × 10^−5^	0.60	11,508
	5	5	2.11 × 10^−5^	0.92	3,659	3.84 × 10^−5^	0.83	3,975
	10	3	1.32 × 10^−5^	0.97	954	1.83 × 10^−5^	0.79	2,825
	20	4	2.54 × 10^−5^	0.90	236	1.23 × 10^−4^	0.69	2,424
	0	4	1.08 × 10^−5^	0.97	3,466	5.31 × 10^−5^	0.40	33,777
Co-ZIF	2.5	6	1.10 × 10^−5^	0.93	8,443	2.67 × 10^−5^	0.99	2,420
	5	4	8.25 × 10^−6^	0.93	6,032	2.37 × 10^−5^	0.67	1848
	10	8	1.22 × 10^−5^	0.94	3,211	1.23 × 10^−5^	0.99	996
	20	8	1.67 × 10^−5^	0.92	2,900	4.59 × 10^−5^	0.96	1,098
	0	6	1.06 × 10^−5^	0.94	7,439	7.11 × 10^−5^	0.41	12,394
Ni-ZIF	2.5	6	1.13 × 10^−5^	0.95	8,705	2.46 × 10^−5^	0.98	2,993
	5	6	1.24 × 10^−5^	0.95	4,901	3.48 × 10^−5^	0.75	4,078
	10	3	1.05 × 10^−5^	0.98	1,089	1.47 × 10^−5^	0.81	2,868
	20	3	1.35 × 10^−5^	0.97	1,001	2.08 × 10^−5^	0.86	1952

Collectively, these observations indicate that the ability of the metal-core ZIFs to occupy the active sites on the metal surface is reduced with increasing ammonia concentration, which disrupts the adsorption of these ZIFs onto the copper metal surface, leading to a reduction in their corrosion inhibition properties.

**FIGURE 9:**
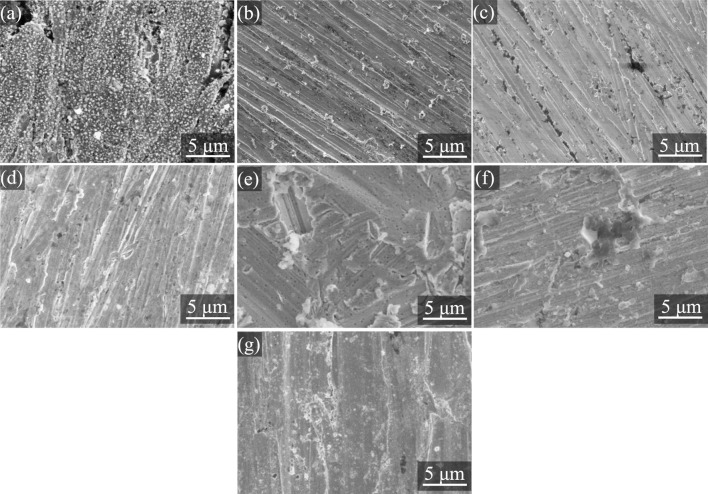
SEM images copper immersed in 0.5 mol/L NaCl solution containing different metal center ions-ZIFs and NH_3_·H_2_O for 24 h at 25 °C **(a)** blank **(b)** Zn-ZIF **(c)** Co-ZIF **(d)** Ni-ZIF **(e)** Zn-ZIF (NH_3_·H_2_O) **(f)** Co-ZIF (NH_3_·H_2_O) **(g)** Ni-ZIF (NH_3_·H_2_O).

Figure 9 presents scanning electron microscopy (SEM) images of copper samples immersed in a blank solution and in a 0.5 mol/L NaCl solution, with the latter either containing ZIFs with different metal centers (Zn, Co, Ni) or ZIFs with different metal centers (Zn, Co, Ni) and ammonia (converted from 20 mg/L) for 24 h. As shown in Figure 9a, the copper surface exposed to the corrosive medium exhibits numerous loose corrosion products, indicating substantial corrosion of the copper substrate. In contrast, following immersion in the same solution with Zn-ZIF, Co-ZIF, and Ni-ZIF (Figures 9b–d), the copper surface appears smooth and uniform, with virtually no visible corrosion products and a noticeable reduction in corrosion. However, compared to the ZIFs-only systems, when the copper samples were immersed in the same solution with ZIFs and ammonia, as depicted in Figures 9e–g, the copper surface transitioned from smooth to somewhat rough, with the formation of some corrosion products, potentially exhibiting features such as pore formation or crystal deformation. These observations suggest that ammonia can alter the structure of ZIFs and interfere with their protective interactions on the copper surface, consequently leading to a degradation in the corrosion inhibition performance of ZIFs.

## Discussion

The morphological integrity of the synthesized Zn-ZIF, Co-ZIF, and Ni-ZIF was assessed using XRD, SEM and TEM, revealing regular and uniform structures. However, increasing ammonia concentration led to progressive degradation of the ZIFs’ structure, ultimately resulting in collapse. FTIR analysis of the ZIFs’ chemical structure in the presence and absence of ammonia showed a diminishing of characteristic ZIF peaks, concurrent with the emergence of peaks indicative of internally coordinated ammonia, suggesting a complex reaction between ammonia and the ZIFs. Electrochemical tests revealed the impact of ammonia on the corrosion inhibition efficiency of ZIFs. Polarization curve analysis demonstrated a significant reduction in the corrosion current density of copper upon the addition of ZIFs in the absence of ammonia. Conversely, an increase in ammonia concentration directly caused a gradual increase in *i*_*corr*_, indicating a reduction in the corrosion inhibition efficiency of the ZIFs, which is linked to the structural damage of the ZIFs. This observation is corroborated by the fitting results of alternating-current impedance spectra. EIS fitting revealed a notable increase in charge transfer resistance and film resistance upon the addition of ZIFs containing different metal center ions, compared to the control. The subsequent addition of ammonia to a 0.5 mol/L NaCl solution containing ZIFs resulted in a decrease in *R*_*f*_ and *R*_*ct*_, indicating that ammonia disrupts the adsorption of ZIFs on the metal surface, thereby diminishing its corrosion inhibition efficiency. Furthermore, SEM imaging of the copper surface post-ammonia addition (specific ammonia concentrations and immersion times should be provided here) revealed an increase in surface roughness, suggesting that ammonia weakens the adsorptive presence of ZIFs on the copper surface, leading to a decline in corrosion inhibition efficiency. These observations collectively suggest that ammonia-induced structural degradation directly compromises the adsorption behavior of ZIFs.

**FIGURE 10:**
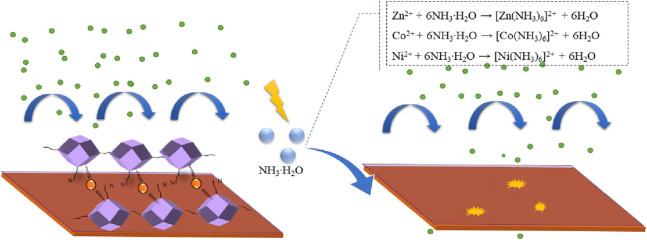
Schematic diagram of the effect of NH_3_·H_2_O on the corrosion inhibition behavior of different metal center ions-ZIFs.

Based on the aforementioned experimental findings, the structural degradation mechanism proposed in Figure 10 is corroborated. ZIFs with different metal center ions are adsorbed onto the copper surface via nitrogen atoms and π electrons on the nitrogen heterocyclic ring within the molecule. This coordination exerts a significant corrosion inhibition effect. However, in the presence of ammonia, gradual complexation with metal ions within the ZIFs occurs, leading to structural collapse. The resultant fragmented molecules fail to provide a protective layer on the metal surface, ultimately resulting in the deterioration of corrosion inhibition efficiency.

## Conclusion

In this study, we immersed ZIFs with Zn/Co/Ni metal centers in an NH_3_·H_2_O environment and monitored their corrosion inhibition efficacy reduction through electrochemical methods. Through a combination of characterization analyses, we explored the underlying mechanisms. The following conclusions can be drawn: In the ammonia aqueous solution, the interaction between ammonia and the metal ions in ZIFs resulted in a corrosion inhibition efficacy reduction. Notably, Ni-ZIF exhibited the most significant performance decay. XRD confirmed initial ZIF crystallinity (Figure 1), while FTIR revealed subsequent structural collapse via metal-ammonia coordination (Figure 4). These findings highlight the critical influence of environmental factors on ZIF stability, suggesting that special attention should be paid to these factors when considering ZIFs for practical applications.

## Data Availability

The raw data supporting the conclusions of this article will be made available by the authors, without undue reservation.
